# Microbiota discovered in scorpion venom

**DOI:** 10.1371/journal.pone.0328427

**Published:** 2026-01-22

**Authors:** Barbara Murdoch, Adam J. Kleinschmit, Carlos E. Santibáñez-López, Matthew R. Graham

**Affiliations:** 1 Department of Biology, Eastern Connecticut State University, Willimantic, Connecticut, United States of America; 2 Department of Natural and Applied Sciences, University of Dubuque, Dubuque, Iowa, United States of America; 3 Department of Biology, Western Connecticut State University, Danbury, Connecticut, United States of America; The University of Lahore, PAKISTAN

## Abstract

With low nutrient availability and presence of numerous antimicrobial peptides, animal venoms have been traditionally considered to be harsh sterile environments that lack bacteria. Contrary to this assumption, recent studies of animal venom and venom-producing tissues have revealed the presence of diverse microbial communities, warranting further studies of potential microbiota in other venomous animals. In this study we used 16S rRNA amplicon sequencing to elucidate whether scorpion venom contained bacteria, to characterize the bacterial communities, and determine if venom microbiomes differed across geologically complex geographic locations. Our study compares the venom microbiome of two scorpion species, sampled from sites in the Mojave and Great Basin deserts, *Paruoctonus becki* (family of Vaejovidae) and *Anuroctonus phaiodactylus* (family of Anuroctonidae), and represents the first assessment of microbial diversity ever conducted using the venom secretion itself, rather than the venom-producing organ and its surrounding tissues.

## Introduction

Microbial communities play essential roles in a wide array of host physiological processes, including digestion [1,2], reproduction [3], and the suppression of other symbionts [4]. Despite their importance, the diversity and potential functions of microbes within animal venom systems remain largely unexplored, e.g., [5]. Animal venom, a complex cocktail of inorganic and organic compounds, serves crucial ecological functions in predator deterrence [6], subduing prey, and even pre-digestion [7], with some evidence suggesting a role in mating [8]. Traditionally, venom has been considered sterile. More recently, studies have begun to unveil the presence of microbiomes within snake and spider venom systems, suggesting a potential influence on infections associated with envenomations [9,10]. This emerging understanding of venom microbiomes underscores the relevance of studying microbial diversity in other venomous animals, such as those found in scorpions.

Scorpions represent an ancient and diverse arachnid lineage comprising nearly 2,900 extant species distributed across a large variety of terrestrial habitats and are renowned for their venom and remarkable resilience to extreme environments. While the majority of scorpion research has focused on systematics [11], venom composition and function (reviewed in [12,13]), and evolutionary origins [14,15], recent efforts have begun to explore their associated microbiomes [16–20]. Early studies targeting the 16S rRNA gene in the gut microbiota of 24 scorpion species revealed significant bacterial diversity across five scorpion families [16,17]. More recently, our team’s work has demonstrated a rich diversity of bacteria, including novel phylotypes of class Mollicutes, within the telson of *Hadrurus arizonensis* and *Smeringurus mesaensis* [18]. Although these prior studies assessed microbes of the gut and telson (where venom production occurs), so far none have characterized the venom microbiota, as this study does.

**Fig 1:**
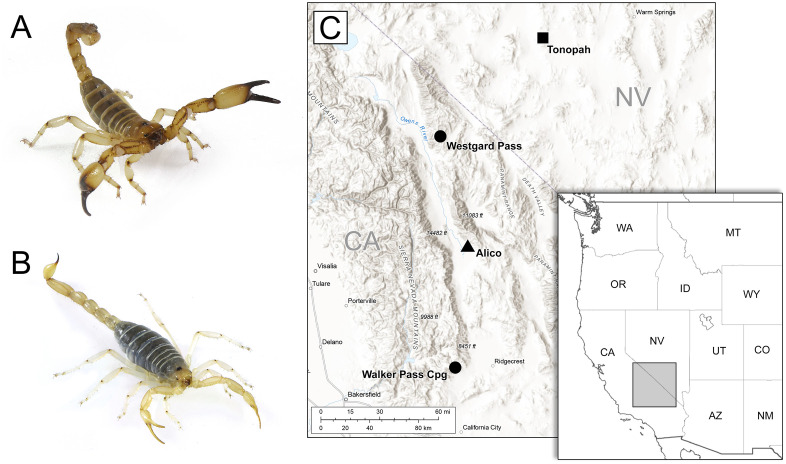
Images of A) *Anuroctonus phaiodactylus* and B) *Paruroctonus becki.* **C)** Topographic map with regional inset map depicting the distribution of collection sites in California and Nevada, USA. Circles mark individual sample locations where only *A. phaiodactylus* was collected. The triangle is where only *P. becki* was collected. Both species were collected from the site marked with a square. The main map uses the USGS National Map “Terrain with Labels” basemap (public domain) as background imagery. An inset map shows the broader regional context using state boundaries from Natural Earth (public domain) created in DIVA-GIS. The authors added state and sampling site labels. All map components are derived from public domain data and are fully compatible with the CC B 4.0 license.

Given that scorpion stings pose a significant global health concern, that secondary infections can complicate envenomation cases [21], and given the vast therapeutic potential of compounds stemming from scorpion venom [22], understanding the microbial communities associated with scorpion venom systems is crucial. While previous studies have identified bacterial genes within the telson and venom glands, the presence and diversity of microorganisms directly within the venom secretion itself remain unknown. To address this gap, we conducted the first assessment of microbial diversity in scorpion venom using two New World scorpion species from distinct families: *Paruroctonus becki* (Gertsch and Allred, 1965) of family Vaejovidae and *Anuroctonus phaiodactylus* (Wood, 1863) of Anuroctonidae. We sampled sites in the Mojave and Great Basin deserts where both species are distributed, but have contrasting ecological preferences: *P. becki* is primarily a psammophilous species often inhabiting alkali-sink environments [23], whereas *A. phaiodactylus* is a pelophilous species burrowing in sedimentary hillsides [24] (Fig 1). Using 16S rRNA amplicon sequencing, we aimed to determine whether scorpion venom contains bacteria, to characterize those bacterial communities, and to assess whether venom microbiomes differed by geographical region.

## Materials and methods

### Scorpion and venom collection

Scorpion collection – We conducted nocturnal field collections using ultraviolet light [25] to obtain 16 *A. phaiodactylus* (3 males, 13 females) and 20 *P. becki* (sex undetermined) specimens from four distinct localities (Permit Number - GW-210220004-21022-001, California Department of Fish and Wildlife). These sites form a latitudinal transect across the ecotone between the Mojave and Great Basin deserts (Fig 1), a region encompassing the Eastern California Shear Zone (ECSZ). The region is a complex, active system of dextral strike-slip faults situated proximal to the San Andreas Fault system, and represents a significant geomorphological driver influencing the distribution and structuring of regional biodiversity patterns. Its long-term influence on topography, hydrology, and habitat fragmentation has been hypothesized to shape evolutionary and ecological processes in various taxa, including *P. becki* [26] and *Aphonopelma* tarantulas [27].

Venom collection – Prior to venom collection, scorpions were sterilized in a laminar flow hood under ultraviolet light for 15 minutes and the telson was additionally sterilized with 70% ethanol, then air dried. Three strategies were used for venom collection. First, scorpions were agitated while enclosed in a plastic bag that was sterilized inside and out with 70% ethanol and air dried. After stinging through the bag onto its outer surface, the venom was retrieved using a micropipette with sterile tip. In the next two strategies, with their aculeus placed into a sterile 1.5 ml Eppendorf tube through ethanol-sterilized parafilm, the scorpion was physically agitated with sterile forceps to elicit venom release. The final strategy involved electrical stimulation using a square wave stimulator of the telson set at 3 events per second, with the duration set to 25 milliseconds and an amplitude of 4 volts. The venom was spun down and DNA was extracted immediately or after storage at −20°C. To control for bacteria on the exterior of the scorpion, 300 µl of Tissue and Cell Lysis Buffer with 50 µg Proteinase K was dripped over the sterile scorpion tails or telsons. The collected liquid wash was transferred into sterile tubes for DNA extraction. These samples termed “*P. becki* exterior controls” underwent 16S PCR prescreening and the resulting amplicons were sent for Illumina sequencing.

### DNA extraction from scorpion venom

DNA was extracted using the methods from Shimwell et al., 2023 [18]. Briefly, a modified Master Pure DNA Purification (Biosearch Technologies) protocol was used to extract DNA from the scorpion venom. Venom volumes ranged from about 3 µl to 20 µl per sample. Venom was thawed on ice, mixed with 300 μl Tissue and Cell Lysis Buffer and 50 μg Proteinase K. Samples were resuspended and incubated overnight in a 56°C shaking water bath. Samples were iced and mixed vigorously with 175 μl of MPC Protein Precipitation Reagent, and centrifuged at 10,000 x g for 10 minutes at 4°C. Supernatants were transferred to fresh tubes and mixed with one volume of cold −20°C isopropanol plus 400 ng glycogen and stored at −20°C for 60 minutes to overnight. Samples were warmed to 4°C and centrifuged at 10,000 x g for 10 minutes at 4°C to pellet the DNA. Supernatants were decanted, and the pellets were washed twice with 70% ethanol. The pellets were air dried for 30 minutes and resuspended in 40 μl of TE buffer (10 mM Tris, 1 mM EDTA, pH 8). To assess potential contamination of our DNA extraction reagents and TE buffer, negative controls included samples with no starting material (venom) that were processed through all steps of the DNA extraction protocol. None of these negative controls produced amplicons during the 16S PCR prescreening.

### Prescreen for 16S rRNA gene from scorpion venom

Venom DNA was prescreened for the 16S rRNA gene indicative of the presence of bacteria [28] using PCR with the 27FHT (5’AGR GTT TGA TYM TGG CT3’) and 1492RHT (5’ GGY TAC CTT GTT ACG3’) primers. Cycling conditions were as follows: 1 cycle 96°C, 5 minutes; 35 cycles 96°C 1 minute, 58°C 1 minute, 72°C 1 minute 50 seconds; 1 cycle 72°C 10 minutes. Final concentrations were 10–50 ng DNA template, 0.5 μM forward and reverse primers, 200 μM dNTPs, 2.5 units Taq DNA polymerase, 20 mM Tris-HCl (pH8.4), 50 mM KCl buffer, 1.5 mM MgCl.

### Illumina sequencing

Illumina sequencing was performed at the Microbial Analysis, Resources, and Services Center, University of Connecticut. The V4 hypervariable region of the 16S rRNA gene was amplified using the 515F (5’-GTGCCAGCMGCCGCGGTAA-3’) and the 806R (5’GGACTACHVGGGTWTCTAAT-3’) primer set and sequenced with Illumina adapters and dual 8 basepair indices [29] on the MiSeq using v2 (2 × 250 bp) paired-end sequencing platform (Illumina). Raw sequence reads were deposited in the National Center for Biotechnology Information (NCBI; accession PRJNA1358989).

### Bioinformatics analysis

#### *P. becki* and *A. phaiodactylus* venom microbiome analysis.

Demultiplexed fastq sequences were obtained after sequencing from the sequencing center. The quality of the raw sequence reads were assessed by a combination of FastQC [30] and MultiQC [31] software. Bioinformatics analysis was performed using the QIIME2 pipeline version 2023.5 [32]. Descriptive statistics and sequence quality information were further reviewed after demultiplexed raw FASTQ files were imported into QIIME2. Based on the raw sequence quality statistics, raw reads were truncated at bp position 191 to remove low quality base calls and the DADA2 software package was used to trim (191 bp), filter, merge paired reads, remove chimeras, and dereplicate sequences [33]. The resulting reads were screened for putative contaminants using the decontam package in R [34] Putative contaminants were identified using the package’s combined method, which integrated the frequency of each ASV relative the total sample DNA concentration and the prevalence of ASVs in negative sequence-run controls. Five flagged ASVs were removed. To further reduce noise from singletons and rare amplicon sequence variants (ASVs), ASVs that composed of <0.1% of smallest sample (~9,700 reads) were filtered. To empirically select a rarefaction depth Good’s coverage was calculated. After evaluating several candidate depths, a depth was selected that minimalized sample loss, exhibited a median Good’s coverage > 95% across samples, and when observed richness approached a plateau with sequencing depth to ensure representation of sample diversity. A sampling depth of 2,100 sequences, which retained 90% of the samples, was used for alpha and beta diversity analysis. The resulting rarefied feature table and ASVs were used to generate a phylogenetic tree. ASV taxonomy was assigned using a pre-trained Naive Bayes taxonomic classifier specific to the V4 region (515F-806R) of the 16S rRNA gene using the Greengenes 2 database [35].

Amplicon data were transformed using R package phyloseq [36] to generate taxon abundance visuals and beta diversity visuals in R (version 3.3.1). Beta diversity was computed using the Weighted Unifrac distance and visualized through Principal Coordinate Analysis (PCoA).

Core Microbiome – A composite 2,100-rarefaction depth *P. becki* and *A. phaiodactylus* feature table was split by scorpion species using QIIME2. The composite and split dataset core microbiomes were analyzed independently using phyloseq (v1.34.0) [36] and microbiome (v1.23.1) [37] software packages for R programing language (v4.3.0). These software packages can be accessed via https://github.com/joey711/phyloseq and https://github.com/microbiome/microbiome respectively. Differential abundance was analyzed using ANCOM-BC [38].

#### *P. becki* exterior control vs. venom microbiome analysis.

A large subset of the *P. becki* exterior control samples exhibited low quality Phred scores at the 3’ ends, thus due to a lack of overlap between forward and reverse reads after truncation at 80 bp, only forward sequences were used for exterior control vs. venom analysis. After forward single-end sequence truncation at 80 bp of samples used in this analysis via DADA2 in the QIIME2 pipeline outlined above, the samples were rarefied to a depth of 17,943 sequences.

### Statistical analysis

Differences between alpha diversity indices were tested using the Kruskal–Wallis test (QIIME2). Beta diversity metric distance was statistically tested by non-parametric multivariate ANOVA (PERMANOVA) with 999 permutations using QIIME 2 software package.

## Results

We used Illumina 16S rRNA amplicon sequencing to determine whether scorpion venom contains bacteria, and to characterize the bacterial communities. We sampled venom from two scorpion species, *Anuroctonus phaiodactylus* (n = 16 scorpions) and *Paruroctonus becki* (n = 20 scorpions) collected from various regions encompassing the Eastern California Shear Zone in California and Nevada, that is known as a driver of regional biodiversity (Fig 1). Some samples were run in duplicate on separate sequencing runs, *A. phaiodactylus* (n = 31 samples sequenced;15 duplicates) and *P. becki* (n = 23 samples sequenced; 9 duplicates;). See S1 File for a list of the samples sequenced. After processing for quality filtering, trimming, chimera removal, *etc*., we generated 2,528,745 16S rRNA paired-end sequence reads, 1,679,193 from *A. phadioactylus* and 849,552 from *P. becki.*

To validate that our sequence reads were in fact sourced from the venom rather than the environment, we compared the bacterial sequences detected in the venom of *P. becki* versus paired exterior controls (n = 6 for each). Beta diversity metrics visualized via Bray Curtis principal component analysis plots showed differences in the microbial composition of the exterior controls compared to the venom (p < 0.006; S1 Fig). Similar results for beta diversity were reached using weighted Unifrac (p < 0.036) and Jaccard (p < 0.004), but not unweighted Unifrac (p < 0.118; S1 Fig). Collectively, these results suggest that the amplicon sequence variants (ASVs) identified in the venom were not due to environmental contamination and that the differences detected were driven largely by differential relative abundance.

Alpha rarefaction plotting was used to assess a sufficient sequencing depth to represent sample diversity (S2 Fig). To include as many samples as possible while covering the majority of the detected diversity, we chose a depth of 2,100 sequence reads for downstream assessment of ASVs, including alpha and beta diversity. After processing, these sequence reads were assigned to 1,841 total ASVs; 159 ASVs (8.6%) were shared between the venom of both scorpion species. Of the 1,325 total ASVs for *A. phaiodactylus,* 88% were unique, whereas 69% (total 516 ASVs) were unique to *P. becki* (S3 Fig).

### Bacterial diversity of scorpion venom

**Fig 2:**
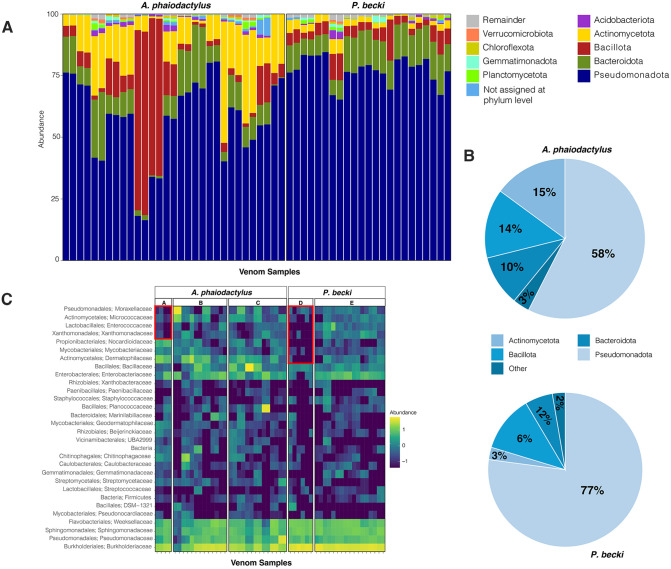
Taxonomic relative abundance profiles at the phylum and family level for *A. phaiodactylus* vs. *P. becki* venom microbiome samples. A) 100% stacked bar plot of top 10 phyla; **B)** Pie charts summarizing percentage abundance for *A. phaiodactylus* and *P. becki*. **C)** Heatmap of top 10 family taxa. Geographical regions include: A, E – Tonopah, NV; B – Walker Pass Campground, CA; C – Westgard Pass, CA; D – Alico, CA.

The ASVs were assigned taxa using the Greengenes 2 database [35]. The most relatively abundant phyla for *A. phaiodactylus* vs *P. becki* respectively, were Pseudomonadota (58% vs 77%), Actinobacteriota (15% vs 3%), Bacillota (14% vs 6%), Bacteroidota (10% vs 12%), and “Others” that includes phyla each with reads of less than 1% (3% vs 2%; Fig 2A, B). A heatmap at the family level shows similar relative abundances between samples for *A. phaiodactylus* and *P. becki* except for the 4 and 7 taxa at the top of the heatmap of columns A and D for *phaiodactylus* and *P. becki,* respectively, which appear to have lower relative abundances (Fig 2C). These samples were collected from the regions of Tonopah, NV, for *A. phaiodactylus* and Alico, CA, for *P. becki,* suggesting a possible link between a region and the venom microbiome.

**Fig 3:**
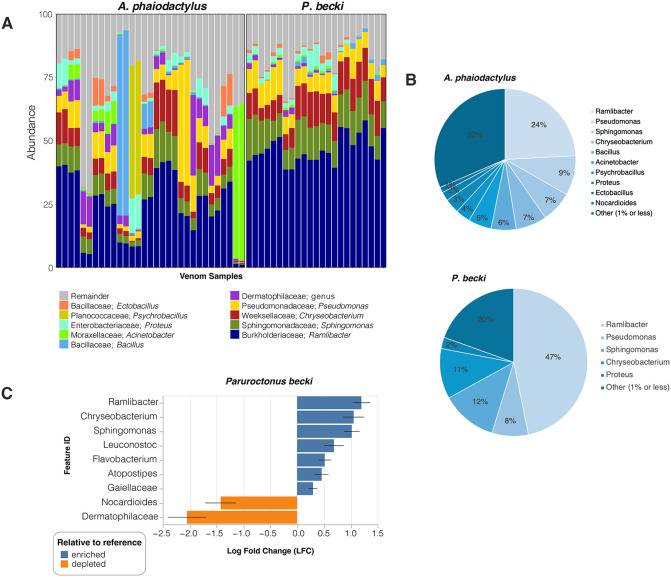
Taxonomic profiles at the genera level for *A. phaiodactylus* vs. *P. becki* venom microbiome samples. A) 100% stacked bar plot of top 10 genera; **B)** Pie charts summarizing percentage abundance for all *A. phaiodactylus* and *P. becki* samples. **C)** ANCOM-BC differential abundance log fold change (LFC) for genera of *A. phaiodactylus* and *P. becki*. Reported taxa exhibit significantly different abundances (Holm adjusted p < 0.05) between the two scorpion species.

For the genera in each venom microbiome (Fig 3A), we found a higher percentage of reads in *P. becki* compared to *A. phaiodactylus* for *Ramlibacter* (47% vs 24%), *Sphingomonas* (12% vs 7%), and *Chryseobacterium* (11% vs 7%), and similar percentages for *Pseudomonas* (8% vs 9%) and *Proteus* (2% vs 3%; Fig 3B). Additional genera seen in *A. phaiodactylus* above 1% of the total reads included *Bacillus* (6%), *Acinetobacter* (5%), *Psychrobacillus* (4%), *Ectobacillus* (2%), and *Nocardioides* (1%; Fig 3B). Although *Psychrobacillus* was not detected in *P. becki,* these other genera were, but at relative abundances lower than 1% (Fig 3B). For *P. becki* and *A. phaiodactylus* respectively, 20% and 32% of all reads represented relative abundances of less than 1% (Fig 3B). To assess differentially enriched and depleted microbiota, we performed an analysis of compositions of microbiomes with bias correction (ANCOM-BC, Fig 3C). In *P. becki,* our analyses showed enrichment for *Ramlibacter, Chryseobacterium*, and *Sphingomonas* (p < 0.05), in addition to *Leuconostoc*, *Flavobacterium*, *Atopostipes*, and the family of *Gaiellaceae* (p < 0.01). In contrast *Nocardioides* and members of the family *Dermatophilaceae* were depleted in *P. becki* relative to *A. phaiodactylus* (p < 0.05; Fig 3C).

### The core venom microbiome of *A. phaiodactylus* and *P. becki*

**Fig 4:**
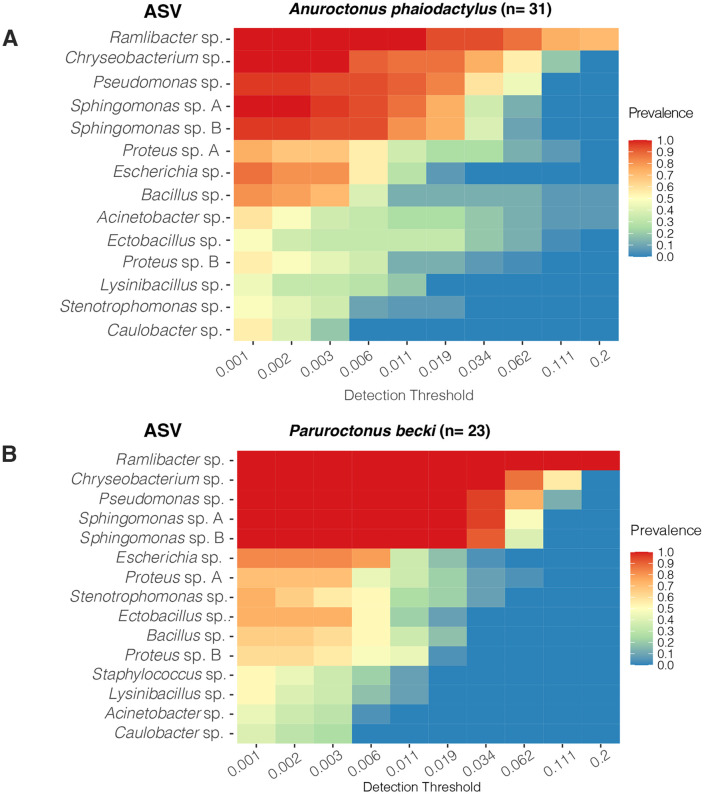
Heatmaps of the abundance–occurrence relationship of the *P. becki* and *A. phaiodactylus* core venom microbial ASVs. **A)** *A. phaiodactylus*, n = 31. **B)** *P. becki*, n = 23. The feature table was rarefied to a depth of 2,100 sequences. Minimal thresholds of 0.01% for relative abundance and 50% for occurrence were applied. Taxonomy naming convention indicates the ASV-associated genus.

We further investigated the taxonomic composition of the venom microbiome in the two scorpion species by focusing on identifying their core microbial constituents. The core microbiome was defined using two conservative criteria: (1) a relative abundance threshold of 0.01% and [32] an occurrence in at least 50% of the samples. These thresholds were selected to strike a balance between capturing ecologically relevant, low-abundance taxa and minimizing noise [39], especially given the rarefaction depth of 2,100 reads. S1 Table shows the number of core microbiome ASVs and their fraction of the total number of ASVs identified at different levels of occurrence. At the ASV level and 50% occurrence threshold, the core microbiome represented a total diversity comprising 1.4% and 4% of the detected genera in *A. phaiodactylus* and *P. becki*, respectively (S1 Table). While the broader microbial communities in the venom were heterogeneous, a relatively stable and tightly aligned core set of microbial taxa emerged independently in both species. ASVs with taxonomy assigned to the genera *Ramlibacter*, *Chryseobacterium*, *Pseudomonas*, and *Sphingomonas* dominated the core microbiome (Fig 4A, B). Of the 159 shared ASVs independent of occurrence between *P. becki* and *A. phaiodactylus* (S3 Fig), 20 formed the shared ASV level core microbiome (≥ 50% occurrence).

### Microbial community diversity in *A. phaiodactylus* and *P. becki*

**Fig 5:**
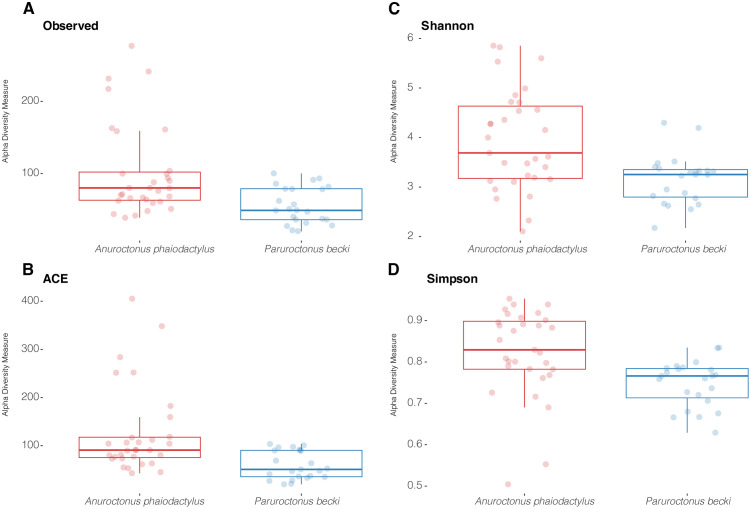
Alpha diversity box plots of *A. phaiodactylus* vs. *P. becki* venom microbiome samples. Species richness was inferred using **A)** Observed taxa and **B)** ACE diversity metrics, while **C)** Shannon, and **D)** Simpson diversity metrics were used to look at how differentially weighting richness and evenness influenced diversity. Pairwise Kruskal-Wallis statistical testing indicated significance for all measures (p < 0.05).

Alpha diversity was performed to determine the within species taxa variation in each scorpion venom bacterial community. For the venom taxa found in *A. phaiodactylus* and *P. becki,* respectively, richness was assessed using Observed taxa (mean ± standard error) 102 ± 11; 56 ± 5, and ACE diversity 125 ± 16; 60 ± 6, that indicated significant differences in alpha diversity between species for both measures (Fig 5A, B; S2 Table, p < 0.05). Differences in Shannon 4 ± 0.2; 3 ± 0.003 and Simpson (0.8 ± 0.02; 0.8 ± 0.01 diversity, for *A. phaiodactylus* and *P. becki,* respectively, were also statistically significant for the venom microbiome in each scorpion species (Fig 5C, D; S2 Table, p < 0.05).

Additional metrics of alpha diversity showed significant differences in the venom microbiota for both scorpion species (S2 Table; p < 0.05). For example, for *A. phaiodactylus* and *P. becki,* respectively, Faith’s Phylogenetic Diversity (mean ± standard error) was 9.15 ± 0.78 and 6.48 ± 0.40); Pielou’s Evenness was 0.61 ± 0.02 and 0.5 6 ± 0.01.

**Fig 6:**
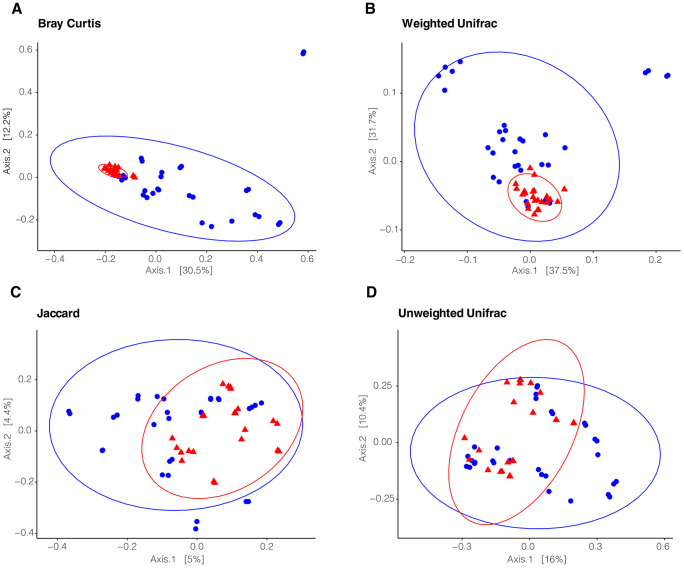
Beta diversity 2D PCoA ordination plots of *A. phaiodactylus* vs. *P. becki* venom microbiome samples. Beta diversity was examined using **A)** Bray Curtis, **B)** Weighted Unifrac, **C)** Jaccard, and **D)** Unweighted Unifrac. Pairwise statistical testing for all distance metrics was significant (PERMANOVA, 999 permutations, p < 0.001).

Beta diversity measures of Bray Curtis, weighted Unifrac, Jaccard, and unweighted Unifrac, all showed statistical significance (Fig 6A–6D; PERMANOVA, p < 0.001), indicating different microbial communities found within the venom of *A. phaiodactylus* compared to *P. becki*. Measures relying on presence/absence, like Jaccard and unweighted Unifrac, appeared to have some overlap, indicating some similarities, rather than clear distinctions (Fig 6C, 6D). In contrast, measures relying on relative abundance such as Bray Curtis and weighted Unifrac, appeared more distinct in space (Fig 6A, 6B). These indices suggest that the observed beta diversity is driven by relative abundance rather than presence/absence.

### Geographical location affects the venom microbiome

We collected scorpion samples from different locations in California and Nevada (Fig 1) and tested whether geographical location influenced their venom microbiomes.

**Fig 7:**
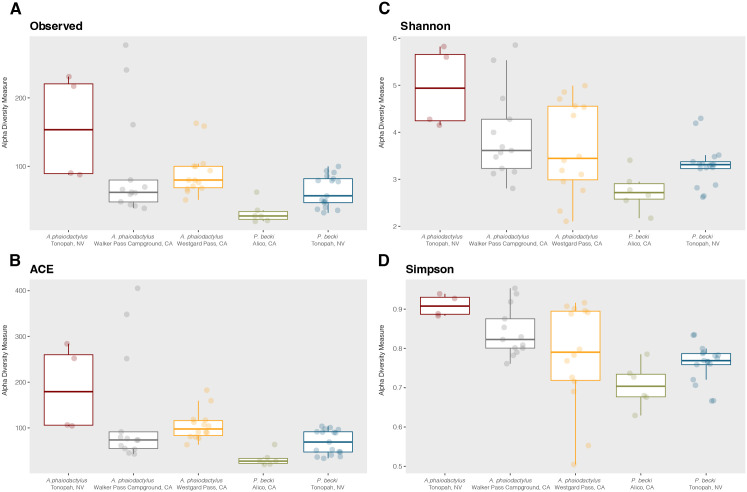
Alpha diversity box plots of *A. phaiodactylus* vs. *P. becki* venom microbiome split by geographical location. Species richness was inferred using **(A)** Observed taxa and **(B)** ACE diversity metrics, while **(C)** Shannon, and **(D)** Simpson diversity metrics were used to look at how differentially weighting richness and evenness influenced diversity. For *A. phaiodactylus* vs. *P. becki at Tonopah, NV:* Observed, q = 0.24; ACE, q = 0.0082; Shannon, q = 0.027; Simpson, q = 0.0078.

We found the geographical location to be a significant factor influencing the microbial diversity of both scorpion species (Fig 7). For measures of richness (Observed and ACE) the median in *A. phaiodactylus* was highest in the region of Tonopah, NV, compared to either Walker Pass Campground, CA or Westgard Pass, CA, and Tonopah, NV showed a greater variability in sample diversity (Fig 7A, 7B). Similar results for richness were seen for *P. becki*, where Tonopah, NV compared to Alico, CA had the highest median and greater variability in sample-to-sample diversity (Fig 7A, 7B). For both scorpion species, alpha diversity metrics that account for richness and evenness, like Shannon and Simpson, supported the differential diversity of the Tonopah, NV region compared to the California-based regions (Fig 7C, 7D). Except for Observed taxa, all other metrics (ACE, Shannon and Simpson) were significant (Holm adjusted p < 0.05). Our results suggest that microbiome diversity can be shaped by geographical location, and are potentially influenced by regional environmental factors, habitat, behavior, or host genetics.

## Discussion

This study, the first of its kind to study scorpion venom microbiota, refutes the notion that venom is a sterile environment devoid of bacteria and corroborates the discovery of microorganisms in the venom of other organisms, like snakes and spiders [9,10]. Next-generation amplicon sequencing of scorpion venom from *A. phaiodactylus* and *P. becki* revealed a shared core microbiome, but a significant range of diversity within and between species, the latter being driven by differential relative abundance. Further, venom microbiota showed significantly varying alpha diversity by geographical location, with the Tonopah, NV population in the Great Basin desert, being more diverse than the California locations to the south.

Extracting venom for scientific research presents well-documented challenges, as experienced by our team and others [9,10]. These difficulties stem not only from the need for specialized expertise in safely collecting and handling venomous animals but also from several limiting factors during the extraction process itself. These limitations include the proportion of animals that yield venom, the small volume of venom released per individual—particularly relevant for subsequent DNA extraction—and potential data loss during sequencing and analysis. In our study, we successfully extracted individual venom samples from an average of 92% of our scorpions: 89% (16/18) for *A. phaiodactylus* and 95% (20/21) for *P. becki*. Furthermore, we successfully extracted DNA from all collected venom samples, indicating minimal losses during these initial project phases. Most data losses occurred during the analysis phase. While all *A. phaiodactylus* samples (16/16) advanced through the analysis pipeline, only 70% (14/20) of the *P. becki* samples could be included in the final analysis due to insufficient sequencing reads.

Our finding that geographic location significantly influenced the venom microbiome of both scorpion species, despite limited spatial sampling, aligns with observations across other widespread terrestrial animal groups. Similar to social spiders [40], our results suggest that the scorpion venom microbiome is partially shaped by environmental filtering across distinct geographic sites. Environmental filtering works by having the distinct local abiotic factors (like soil type or regional climate) of each site selectively prevent certain microbial taxa from successfully colonizing and establishing within the host microbiome (reviewed in [41], thereby driving some of the observed compositional differences.

Importantly, strong geographic signals in microbiome datasets have also been observed in other ectothermic animals. Eliades et al. (2022) found that sampling locality and broader biogeographic zones were significantly associated with microbial assemblages in widespread gecko species, particularly *Cyrtodactylus philippinicus* [42]. This consensus across terrestrial invertebrates and reptiles suggests that local environmental conditions may be a strong selective pressure on host-associated microbial communities.

The specific pathway for environmental microbial entry into the venom is yet to be fully determined. However, the external location of the venom pore at the tip of the scorpion telson represents a key interface with the environment, regularly contacting surfaces such as substrate and prey during hunting and movement. This frequent contact provides a plausible route for the environmental filtering signal to manifest within the venom microbiome.

In contrast to the clear effect of geography, Eliades et al. (2022) observed that finer-scale microhabitat variation did not significantly impact the bacterial community structure of the widespread gecko species [42]. This distinction highlights an interesting gap in our understanding of arthropod microbiome assembly. As both *A.* *phaiodactylus* and *P. becki* are fossorial animals, their close association with the substrate may mean their venom microbiome is highly sensitive to the specific microhabitat in ways that are not seen in more mobile reptiles like geckos. Given our limited sampling, the influence of microhabitat was not testable, but we hypothesize that these effects may be highly localized and thus masked by the greater variation observed across the four geographic sites. Future work should implement denser sampling, collecting individuals from distinct microhabitats within a single geographic location to isolate this variable and quantify its effect relative to the geographic signal.

The microbial communities inhabiting metazoans appear to be shaped by a combination of ecological, environmental, and host-specific factors [43]. Environmental context, such as geographic location and habitat type, may influence the presence and composition of distinct microbial taxa, with certain bacteria exhibiting a stronger capacity to adapt to anatomical microhabitats [44]. The study of core taxa may assist in identifying microbial persistence requirements within the habitat [43,45].

The microhabitat of the venom gland, the site of venom production, may select for bacteria with resilience in adaptive ability and functional features that allow establishment after gaining access from the external environment [9,46]. These microcosms in venom-associated tissue are likely to be defined by extreme and selective pressures, including the presence of antimicrobial peptides and low nutrient availability, which likely act as environmental filters that favor microbial taxa with resilience and adaptive functional traits. Found to be differentially enriched in the venom microbiota of *A. phaiodactylus* compared to *P. becki,* was the genus *Nocardioides* (and family Dermatophilaceae), which can endure extreme environments, and are known for their biotransformation capacity, including pollutant degradation and toxin removal, in addition to production of antimicrobial compounds [47]. Interestingly, antimicrobial compounds in animal venoms are presumed to be made by the host, but the discovery of venom-based microbial communities in this study of scorpions and other studies of snakes and spiders, instead validates the notion of antimicrobial production by the inherent bacteria [9,10,18].

The open structure of the scorpion venom apparatus, particularly the ducts exposed near the tip of the aculeus, may offer an access point for opportunistic environmental microbes. However, the successful colonization of this niche likely requires more than passive entry, as microbes must also withstand the antimicrobial components of the venom and potentially exploit protective or symbiotic mechanisms to persist. One genus that exemplifies such adaptability is *Ramlibacter*, which was found to have a 100% prevalence and accounted for 24% and 47% of genera from *A. phaiodactylus* and *P. becki* scorpion species, respectively. Taxonomically placed within the Pseudomonadota phylum (order Burkholderiales, family Comamonadaceae), *Ramlibacter* is known for its high environmental resilience, including tolerance to desiccation, dormancy under nutrient-poor conditions, and persistence in arid terrestrial environments [48]. These conditions closely align with the arid habitats where the scorpions in this study were collected. Interestingly, in our previous study, *Ramlibacter* was not detected in the venom gland of either *H. arizonensis* or *S. mesaensis,* despite that 12% and 44% of their microbiomes (respectively) were Pseudomonadota taxa [18]. In contrast, *Ramlibacter* has been reported in the venom gland microbiome of the spider *Steatoda grossa*, a metropolitan species sourced from captive conditions [49], suggesting that this genus may opportunistically colonize venom glands across diverse arthropod hosts. Furthermore, *Ramlibacter* has been detected in a wide range of terrestrial and aquatic environments worldwide increasing the likelihood of colonization of diverse venomous species [49].

In addition to *Ramlibacter*, we observed 100% prevalence and relatively high abundance of *Sphingomonas*, *Chryseobacterium*, *Pseudomonas,* and *Proteus* across scorpion venom samples. These genera have also been documented in the venom or venom-associated tissues of multiple spider species [10,46,50], highlighting a potential pattern of convergence in venom gland-associated microbiota among distantly related venomous metazoans. *Sphingomonas* and *Chryseobacterium* were also found in the scorpion telson microbiota [18].These findings support the hypothesis that venom glands may serve as selective environments favoring certain taxa with functional traits conducive to survival in such chemically hostile niches.

The potential for microbial ingress through external structures is further supported by examples in other venomous animals. In spiders, opportunistic microbes are thought to colonize the venom glands via external chelicerae, while in snakes, biofilm formation has been implicated in the oral cavity and venom delivery structures [9]. Similarly, scorpions undergo cleaning behaviors before and after feeding, for example to clean their stingers with their chelicerae, or after a sting by rubbing the stinger on a substrate like sand [51]. These behaviors suggest potential microbial entry into the scorpion venom organ through the oral cavity, the external environment, or through captured prey, either after being stung or after being consumed by the scorpion.

Beyond the most dominant core taxa, our study also identified members of the Enterococcaceae family, that includes the genus *Enterococcus*, at or below 0.2% of total reads for both scorpion species, which has previously been isolated from the venom of the black-necked cobra. Notably, *E. faecalis* strains from that study possessed genes associated with enhanced membrane integrity and antimicrobial resistance, which suggests microbes with characteristics beneficial for the venom environment may be selected for [9]. Collectively, these findings raise thought-provoking inquiries about the potential functional roles and genomic adaptations of scorpion-associated microbial taxa.

Other genera detected in our sample core microbiome, including *Stenotrophomonas*, *Acinetobacter*, *Bacillus*, and *Staphylococcus*, have been previously reported in venom-associated tissues of spiders and marine gastropods [9,10,46,52]. For example, *Stenotrophomonas* spp. have been proposed to interact directly with venom peptides, potentially modifying their biological activity. The detection of Caulobacteraceae taxa in our sample core adds further depth to the taxonomic range of scorpion venom gland-associated microbes also detected in other venom-associated tissues [52], warranting future investigation into their ecological and functional roles. The clinical potential of venom compounds suggests the possibility, that microbes within their unique venom microhabitats may provide the necessary chemical precursors for the production of these therapeutic compounds.

Our unique multi-site sampling of venom microbiomes from *A. phaiodactylus* and *P. becki* offers insights into the influence of geographic and geological factors on microbial communities in venoms. All samples were collected within the tectonically complex Eastern California Shear Zone (ECSZ), an area where significant geological activity [53] is presumed to have driven diversification in both *P. becki* [26] and *Aphonopelma* tarantulas [27]. For *P. becki*, our samples encompass two distinct clades as identified by Graham et al. (2013): the White-Inyo Clade (Alico, CA) and the Great Basin Clade (Tonopah, NV). Similarly, our *A. phaiodactylus* samples were collected from three localities within the ECSZ region: the southern Sierra Nevada (Walker Pass Campground, CA), the White-Inyo Mountains (Westgard Pass, CA), and the Great Basin Desert (Tonopah, NV). Unpublished phylogenomic data (Graham) suggest that these *A. phaiodactylus* sites also represent distinct clades. Interestingly, the Tonopah samples from the Great Basin consistently exhibited the highest venom microbiome diversity for both scorpion species (Fig 7). This observation aligns with phylogeographical data indicating that both *P. becki* and *A. phaiodactylus* underwent recent range expansions in the Great Basin Desert, likely while colonizing new desert habitat that became available as climates warmed following the Last Glacial Maximum.

Gut microbiomes can change quickly and predictably alongside host evolution, implying that host-microbe interactions are important drivers of host adaptation and diversification [54,55]. As such, we hypothesize that scorpion venom microbiomes may have diverged concurrently with host clade formation during the Pliocene and Pleistocene. Furthermore, the increased diversity in Great Basin Desert samples may reflect a phenomenon where venom microbiomes become more diverse as their hosts colonize and adapt to novel habitats.

As a species expands its range into new environments, individuals encounter and acquire novel environmental microbes through various routes [56,57], which can lead to an enrichment of their internal microbial communities. This increased diversity may confer an adaptive advantage, as a more diverse microbiome can offer “colonization resistance” against opportunistic or pathogenic microbes [58,59], which could be particularly beneficial in challenging or unfamiliar environments, like new arid habitats that became suitable for colonization in the Great Basin Desert. Furthermore, host adaptation to new habitats can involve co-evolutionary processes with their microbiomes, where selective pressures favor symbionts that expand the host’s abiotic niche and enable colonization of otherwise harsh environments, a process known as biotic facilitation (reviewed in [60]). Thus, the proposed postglacial range expansions of *P. becki* and *A. phaiodactylus*, as well as other co-distributed Great Basin Desert taxa [61–63], provide a temporal framework for such a diversification of their associated venom microbiomes. Integrating phylogeographic datasets for these taxa with more geographically comprehensive microbiome sampling would provide the necessary resolution to formally test the “colonization resistance” hypothesis, making these co-distributed systems ideal model organisms for such an investigation. Therefore, the biogeography of these systems offers an exemplary framework for testing both hypotheses of host-microbiome co-diversification and microbial enrichment during novel habitat colonization.

In conclusion, we identified a potential dual selective regime shaping the venom microbiome. Venom chemical and physiological properties might impose an internal selective filter, resulting in the partial convergence of microbial taxa (core) shared across diverse venomous organisms, which in turn is independent of habitat. Simultaneously, specific environmental and host factors determine the composition of the variable non-core fraction. This pattern suggests that venom secretions represent a unique, underexplored microbial niche influenced both by strong venom-derived selective pressures and host-specific ecological factors. Further comparative studies across scorpion populations and habitats is warranted to shed more light on how geography and ecology may influence the composition of microbial communities.

## Supporting information

S1 Fig Principal component analysis plots of *P. becki* microbiome samples from exterior surface swabs and venom. Ellipses represent 95% confidence intervals for the grouped distance metrics (PERMANOVA, 999 permutations, Bray Curtis, p = 0.006; Jaccard, p = 0.004; Weighted Unifrac, p = 0.036; Unweighted Unifrac p = 0.118).
(PDF)

S2 Fig Rarefaction curves of venom-associated microbiome samples from *A. phaiodactylus* and *P. becki* regarding ASVs. Vertical purple dashed line represents the rarefying sequence depth used for downstream analysis.
(PDF)

S3 Fig Venn diagram of ASV presence/absence for A*. phaiodactylus* and *P. becki* venom microbiome. All ASVs at 2,100 rarefaction level.
(PDF)

S1 Table The number of core microbiome ASVs and their fraction of the total number of ASVs identified at different levels of occurrence in *A. phaiodactylus* (n = 31) and *P. becki* (n = 23) samples. (PDF)

S2 Table Pairwise Kruskal-Wallis statistical test for alpha diversity metrics between *Anuroctonus phaiodactylus* (n = 31) and *Paruroctonus becki* (n = 23). Input samples were rarefied at a depth of 2,100 sequences.
(PDF)

S1 File List of all sequenced scorpion venom samples for *Anuroctonus phaiodactylus* (n = 31) and *Paruroctonus becki* (n = 23) by sample ID, location and sex. (XLSX)
