# Supplementary material for: Microbiota discovered in scorpion venom
Source: PLoS One. 2026 Jan 22;21(1):e0328427. doi: 10.1371/journal.pone.0328427 (PMC12826464; doi:10.1371/journal.pone.0328427)

Figure S1. Principal Component Analysis plots of *P. becki* microbiome samples from exterior surface swabs and venom. Ellipses represent 95% confidence intervals for the grouped distance metrics (PERMANOVA, 999 permutations, Bray Curtis, p=0.006; Jaccard, p=0.004; Weighted Unifrac, p=0.036; Unweighted Unifrac p = 0.118).

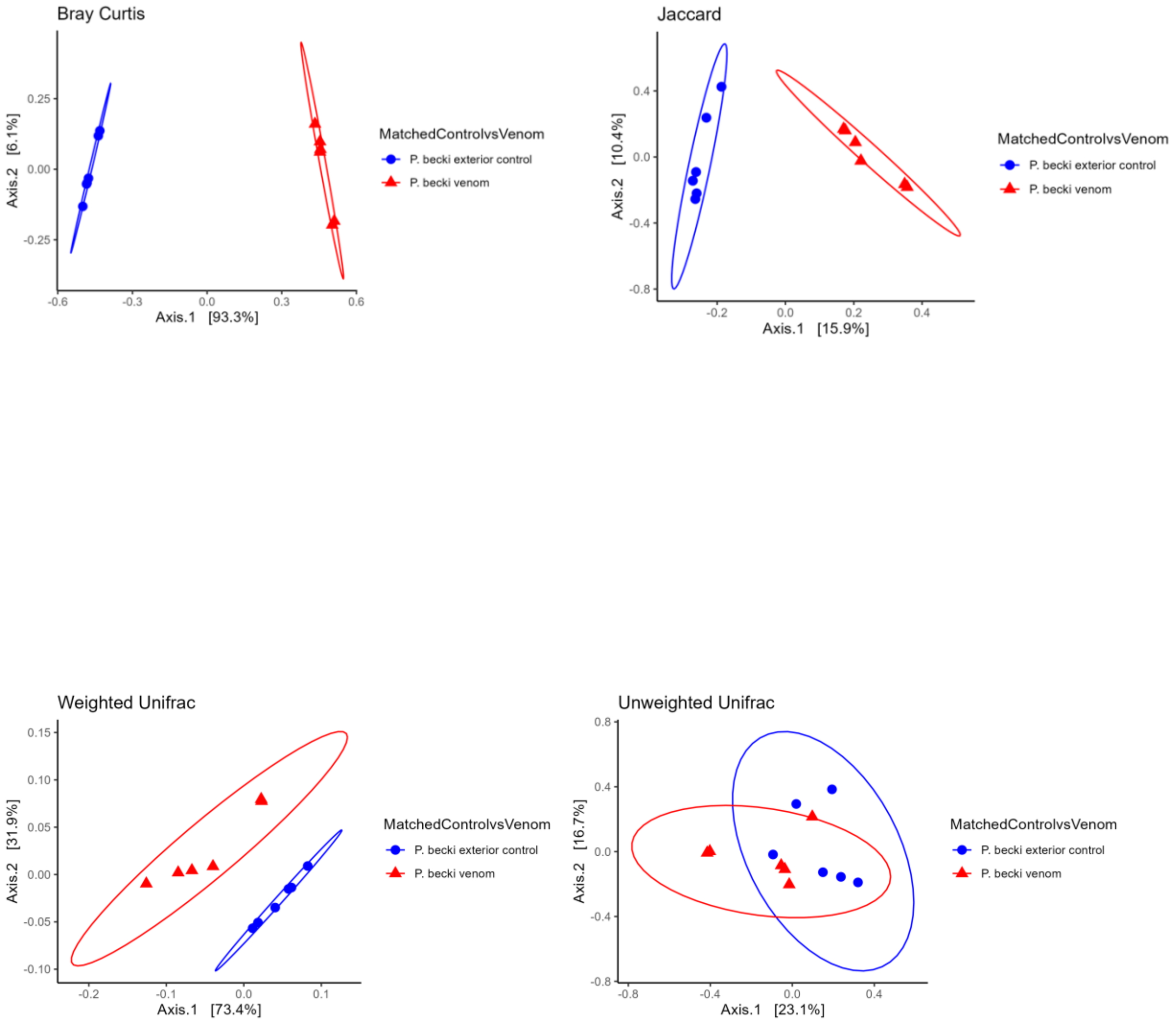

Supplement: S1 Fig — Ellipses represent 95% confidence intervals for the grouped distance metrics (PERMANOVA, 999 permutations, Bray Curtis, p = 0.006; Jaccard, p = 0.004; Weighted Unifrac, p = 0.036; Unweighted Unifrac p = 0.118). (PDF) [file pone.0328427.s001.pdf]
