# Supplementary material for: Microbiota discovered in scorpion venom
Source: PLoS One. 2026 Jan 22;21(1):e0328427. doi: 10.1371/journal.pone.0328427 (PMC12826464; doi:10.1371/journal.pone.0328427)

Figure S2. Rarefaction curves of venom microbiome from *A. phaiodactylus* and *P. becki* regarding ASVs. Vertical purple dashed line represents the rarefying sequence depth used for downstream analysis.

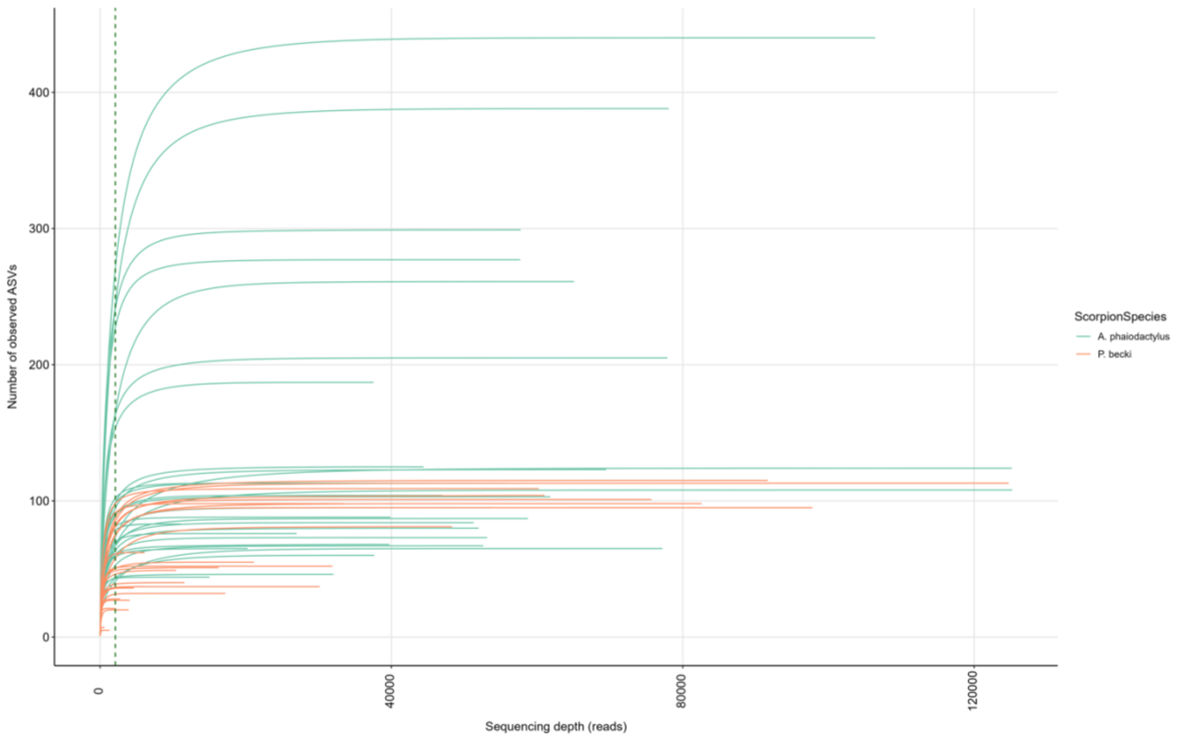

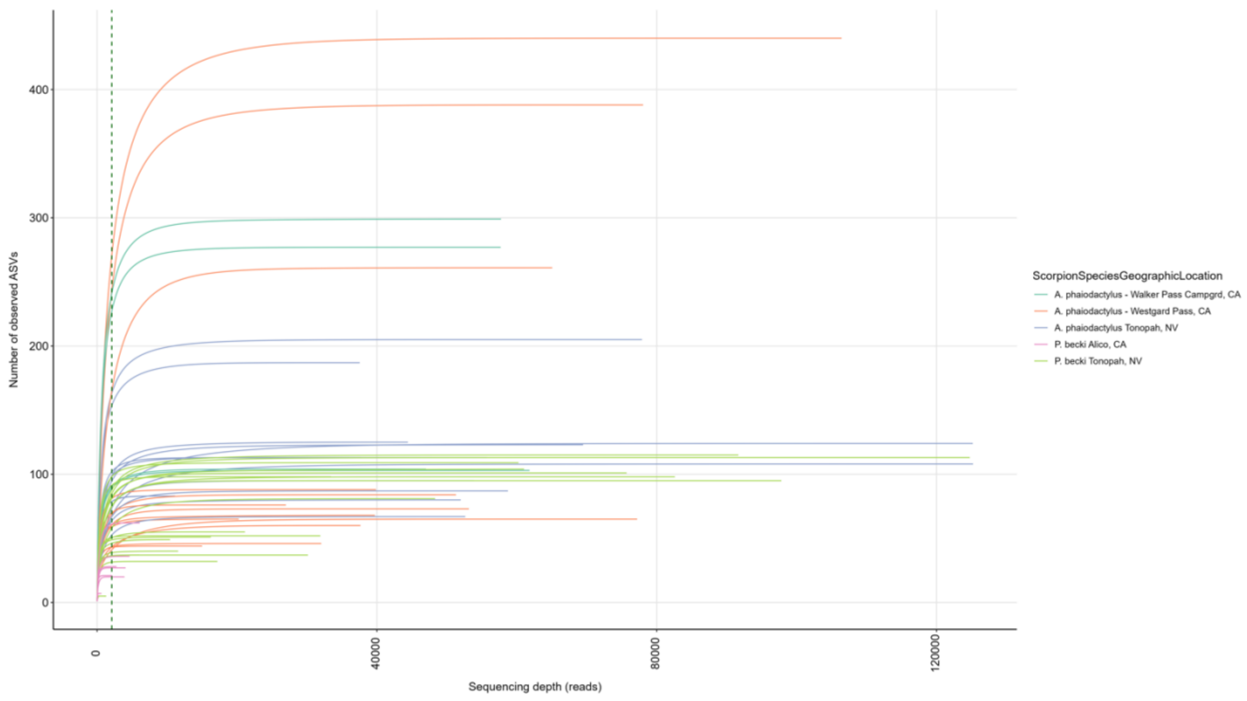

Supplement: S2 Fig — Vertical purple dashed line represents the rarefying sequence depth used for downstream analysis. (PDF) [file pone.0328427.s002.pdf]
