# Supplementary material for: Microbiota discovered in scorpion venom
Source: PLoS One. 2026 Jan 22;21(1):e0328427. doi: 10.1371/journal.pone.0328427 (PMC12826464; doi:10.1371/journal.pone.0328427)

Figure S3. Venn diagram of ASV presence/absence for *A. phaiodactylus* vs. *P. becki* venom microbiome. All ASVs at 2,100 rarefaction level.

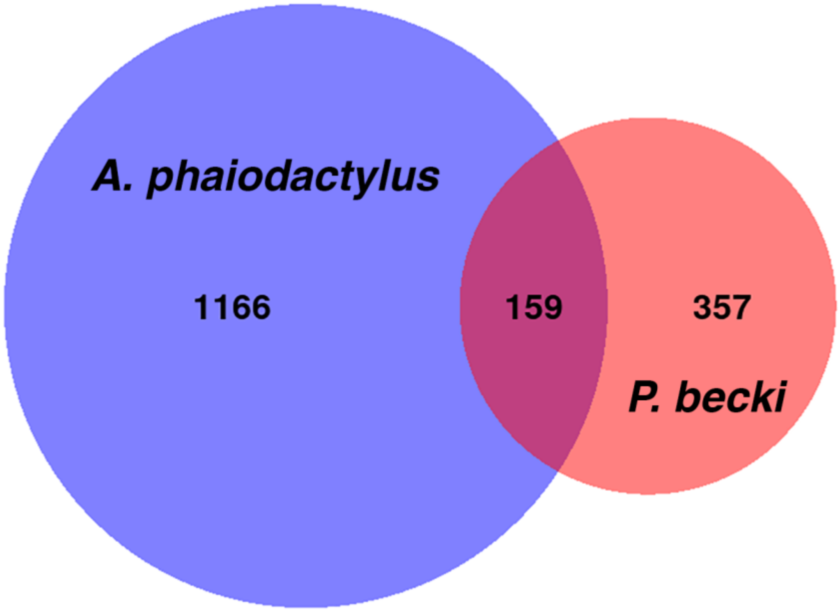

Supplement: S3 Fig — All ASVs at 2,100 rarefaction level. (PDF) [file pone.0328427.s003.pdf]
