# Supplementary material for: Microbiota discovered in scorpion venom
Source: PLoS One. 2026 Jan 22;21(1):e0328427. doi: 10.1371/journal.pone.0328427 (PMC12826464; doi:10.1371/journal.pone.0328427)
Supplement: S1 Table — (PDF) [file pone.0328427.s005.pdf]

S1 Table. The number of core microbiome ASVs and their fraction of the total number of ASVs identified at different levels of occurrence in *A. phaiodactylus* (n=31) and *P. becki* (n=23) samples.

Sample Occurrence Threshold	*A. phaiodactylus*		*P. becki*	
	# of ASVs	Fraction of total ASVs	# of ASVs	Fraction of total ASVs
0.010	1309	1.0000	494	1.0000
0.109	141	0.1077	86	0.1741
0.208	51	0.0390	46	0.0931
0.307	39	0.0298	29	0.0587
0.406	25	0.0191	22	0.0445
0.505	18	0.0138	20	0.0405
0.604	17	0.0130	14	0.0283
0.703	13	0.0099	8	0.0162
0.802	11	0.0084	6	0.0121
0.901	7	0.0053	5	0.0101
1.000	6	0.0046	5	0.0101
