# Supplementary material for: Microbiota discovered in scorpion venom
Source: PLoS One. 2026 Jan 22;21(1):e0328427. doi: 10.1371/journal.pone.0328427 (PMC12826464; doi:10.1371/journal.pone.0328427)
Supplement: S2 Table — Input samples were rarefied at a depth of 2,100 sequences. (PDF) [file pone.0328427.s006.pdf]

S2 Table. Alpha diversity in venom microbiota for *A. phaiodactylus* and *P. becki*
Pairwise Kruskal-Wallis statistical test for alpha diversity metrics between *Anuroctonus phaiodactylus* (n=31) and *Paruroctonus becki* (n=23). Input samples were rarefied at a depth of 2,100 sequences.

**Alpha Diversity Metric**	**Mean (Standard Error)**		**p-value**
	A. phaiodactylus	***P. becki***	
Faith's Phylogenetic Diversity	9.15 (0.78)	6.48 (0.40)	0.0281
Pielou's Evenness	0.61 (0.02)	0.56 (0.01)	0.0382
Observed Features	101.71 (11.43)	55.65 (5.26)	0.0012
Shannon Diversity Index	3.94 (0.18)	3.16 (0.10)	0.0030
ACE	125.16 (16.18)	59.62 (6.06)	0.0002
Chao1	122.58 (15.08)	59.13 (5.94)	0.0003
Simpson	0.82 (0.02)	0.75 (0.01)	0.0003
